# Analysis of molecular inversion probe performance for allele copy number determination

**DOI:** 10.1186/gb-2007-8-11-r246

**Published:** 2007-11-20

**Authors:** Yuker Wang, Martin Moorhead, George Karlin-Neumann, Nicholas J Wang, James Ireland, Steven Lin, Chunnuan Chen, Laura M Heiser, Koei Chin, Laura Esserman, Joe W Gray, Paul T Spellman, Malek Faham

**Affiliations:** 1 Affymetrix Inc., Shoreline Blvd, South San Francisco, CA 94080, USA; 2 LBL 1 Cyclotron Rd, MS977R225A, Berkeley, CA 94720, USA; 3 Comprehensive Cancer Center, Sutter Street, University of California San Francisco, San Francisco, CA 94143, USA

## Abstract

We have developed a new protocol for using molecular inversion probes to accurately and specifically measure allele copy number. The new protocol provides for significant improvements, including the reduction of input DNA (from 2 μg) by more than 25-fold (to 75 ng total genomic DNA), higher overall precision resulting in one order of magnitude lower false positive rate, and greater dynamic range with accurate absolute copy number up to 60 copies.

## Abstract 2

A new protocol for using molecular inversion probes to specifically and accurately measure allele copy numbers.

## Background

Chromosomal copy number analysis has been important in the study of tumor samples for decades. Changes in copy number have already been demonstrated to predict patients' response and/or prognosis [1], which gives hope that this can be applied in large scale to significantly affect clinical care in the future. In order to fulfill this promise, technologies that are able to assess copy number on the whole genome scale in a large number of samples are required. Since the development of comparative genomic hybridization (CGH) [2], many technologies have been developed to address this need. These include bacterial artificial chromosome (BAC) CGH and, more recently, CGH employing several types of oligonucleotides arrays [3-7]. Some of the newer CGH methodologies allow for allelic information to be obtained [4,5,7,8]. The utility of measurement of allele copy number (ACN) includes the identification of loss of heterozygosity (LOH) events [4] and the allelic composition at amplified loci [9].

One of the techniques that have previously been described for the measurement of ACN is molecular inversion probes (MIPs) [10-12]. Briefly, MIP probes are circularizable oligonucleotides, where the two ends carry two sequences that are complementary to two sequences on the genome separated by one nucleotide (exactly where the variant to be genotyped is). After hybridization to the genomic DNA, the reaction is split into four tubes where a single nucleotide is added to each tube. Upon the addition of the nucleotide, the MIP probe is ligated closed (but this only occurs in the tube with the nucleotide that is complementary to the allele on the genome), turning the probe into a circle. This structure can be selected for by the use of exonucleases, allowing for minimal 'cross talk' between probes and making it possible to obtain high quality data from highly multiplexed assays (>50,000-plex). Ultimately, these products are amplified and hybridized onto an Affymetrix microarray to identify the present products.

The MIP assay differs from other highly multiplexed (tens of thousands to hundreds of thousands) genotyping techniques in that it utilizes enzymatic steps in solution to capture specific loci, which is then followed by an amplification step. Such a combination of enzymatic steps confers a high degree of specificity on the MIP assay. The high specificity and minimum 'cross talk' between loci or alleles results in precise measurements as well as large assay dynamic range. In addition, the amplification of the loci of interest only simplifies the task of detection and provides the ability to use lower amounts of input genomic DNA. The high precision, large dynamic range, and low DNA usage are demonstrated in this study. Finally, because MIP requires only 40 base-pairs of intact genomic DNA, its use in degraded samples, such as formaldehyde fixed paraffin embedded samples, may offer distinct advantages.

We have made significant advancements in this technology. As a result, the false positive rate has decreased by an order of magnitude and the dynamic range extended to achieve accurate absolute copy number measurements up to 60 copies, while reducing the input genomic DNA requirement by more than 25-fold.

We describe the performance of the MIP assay using several types of metrics that are broadly useful to all copy number assays: the ability to discriminate a copy number aberration from normal at the total as well as ACN level; and the ability to accurately quantify the level of copy number aberration at both the total and ACN levels.

## Results

### MIP copy number assay modification

We have previously described the use of MIP for copy number analysis [11,12]. We have now improved the performance of the technology through modifications of the MIP copy number protocol and through improved data analysis. The improved performance allows ACN data to be obtained using 75 ng of human genomic DNA.

The first implementation of the MIP ACN assay required 2 mg of genomic DNA. We discovered that only a fraction of the genomic templates hybridized to MIP probes that are then circularized and amplified. We hypothesized that increasing the number of MIP molecules and decreasing the hybridization volume should increase the number of MIP molecules bound to their genomic targets. We tested this hypothesis and verified that increasing the number of MIP molecules by a factor of four and decreasing the hybridization volume (from 27 ml to 6.7 ml) allowed us to substantially decrease genomic DNA input. After the hybridization, buffer is added to increase the volume to 27 ml, and the rest of the protocol is unmodified.

In the standard genotyping protocol, the genomic target is split into four reactions, where one of each of the four nucleotides is added. We recognized that we could decrease DNA input requirements by performing a smaller number of these reactions We reasoned that if we were to use only one set of single nucleotide polymorphisms (SNPs; for example only the most common C/T SNPs), we would decrease the DNA requirement by 50%. Similarly, adding two nucleotides into each of two reactions leads to the same result. We have implemented this variant protocol by adding G and C nucleotides into one tube, and adding A and T into another. In this scenario, about 85% of SNPs in the human genome (all but G/C and A/T SNPs) can be assessed. An advantage of decreasing the number of reactions is that it requires only two independent readouts rather than four (that is, four colors on one array or one color on four arrays). In the optimized procedure, 75 ng of genomic DNA are mixed with more than 50,000 probes in a small volume (6.7 ml). The hybridized probe:target genomic DNA are split into two reactions, where two nucleotides are added to each of the two tubes. The two reactions are processed separately and read on two independent arrays, which was found to yield better data than two colors on one array (data not shown).

One effect that requires correction in quantitative assays on arrays is the phenomenon of saturation. This is especially important for correct estimations of amplifications. We have implemented a Langmuir correction for the non-linear relationship between signal and copy number [13]. Our algorithm was developed on a separate data set, and the data shown here is an independent set. Using this algorithm we have been able to measure copy number in a linear fashion at levels over 60 copies (see below).

### Detection of aberrations

An important aspect of the copy number performance is the detection of aberrations where the copy number is distinct from 2. The degree of discrimination between copy number 2 and the aberrant copy can be understood through receiver operator characteristic (ROC) curves showing the trade off between false positive rate and sensitivity (1 - false negative rate) given data on regions with known copy number. The presence of cell lines carrying 1, 3, 4, or 5 X chromosomes provides a good resource for the study of the performance of the technology in this copy number range [2]. For example, in the assessment of cell lines with one X chromosome (males) one can make a threshold at copy number 1.5 and any marker on the X chromosome with a copy number below 1.5 would be considered a true positive, and any autosomal marker with a copy number below 1.5 is considered a false positive. By plotting this trade off between true and false positives at many thresholds between copy numbers of 0 to 3, the full ROC curve is generated.

**Figure 1:**
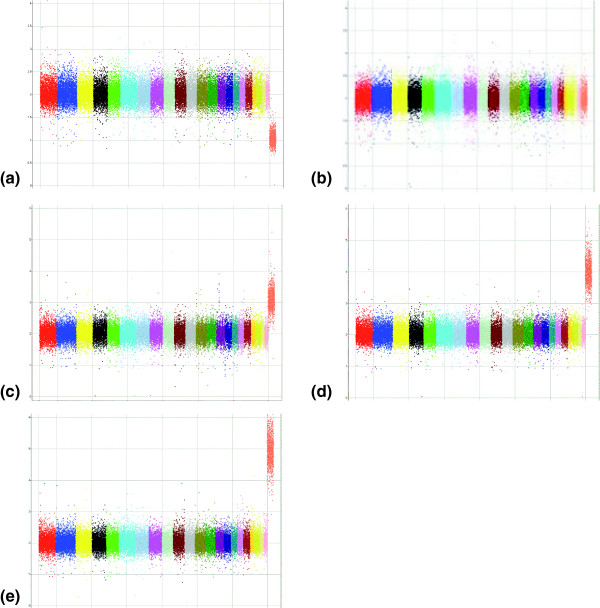
Genomic view of samples with 1-5X chromosomes. The X axis shows the markers in a genomic order, with each chromosome uniquely colored. The Y chromosome depicts the measured copy number for each marker in linear scale. The X chromosome is the last chromosome on the right and is shown in orange. **(a) **A male sample with 1X chromosome. **(b) **A female sample with 2X chromosomes. **(c) **A cell line with 3X chromosomes. **(d) **A cell line with 4X chromosomes. **(e) **A cell line with 5X chromosomes.

**Figure 2:**
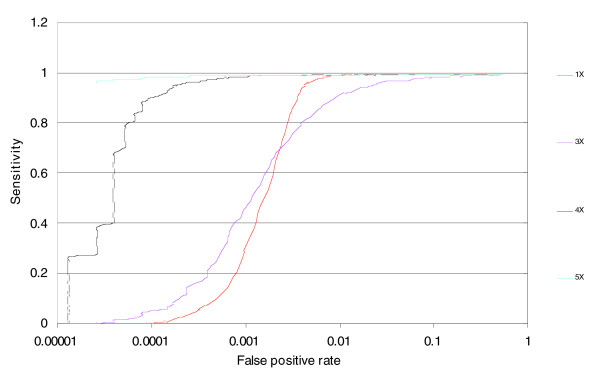
ROC analysis. The x-axis is the rate of false positives (in log_10_), computed as the proportion of autosomal markers that have copy number below any given threshold (for the 1X calculation). The y-axis depicts sensitivity, defined as the proportion of X chromosome markers that have copy number values below the same threshold (for the 1X calculation). The curve is generated by calculating these values at many different thresholds. The curves from the 3X, 4X, and 5X cell lines were generated in an analogous fashion.

To assess the ability of MIP to detect copy number aberrations we used a probe panel containing approximately 53,000 SNPs. We utilized this pool to assay 63 samples (45 unique, 9 duplicate) from the 3 major populations used in the HapMap project. Out of the 53,341 SNPs, 50,806 had genotyping call rates of greater than 90%. We then sorted the remaining SNPs based on the standard deviation of their predicted copy number. We selected the most robust markers for detailed study of copy number performance by selecting those with a standard deviation of less than 12%. This yielded a population of 39,785 markers. Figure 1 shows the copy number estimates across the genome for the different samples carrying one to five copies of the X chromosome. By assuming that males have only one copy of the X chromosome markers and two copies of autosomal markers, we generated ROC curves to describe the trade off between false positive rate and sensitivity for distinguishing one copy from two copies (Figure 2, red line). Similar ROC curves can be generated for the discrimination between 2 and 3, 4, or 5 copies (Figure 2). Comparing the generated ROC curves with our published data for the previous MIP protocol, we find a dramatic improvement. For example, at the same 50% sensitivity level, we found a reduction of the false positive rate by an order of magnitude.

**Figure 3:**
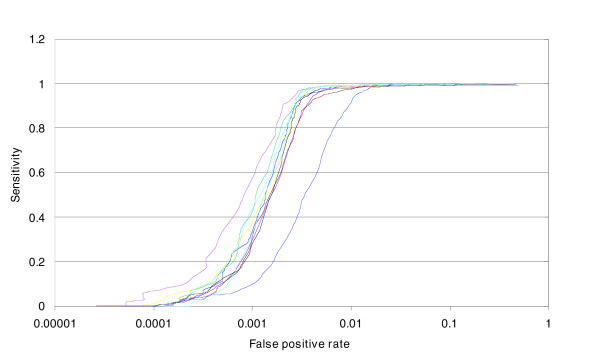
ROC analysis for individual samples. The x-axis is generated in the same fashion as Figure 2, except that the curve for each sample is plotted separately. The average curve is the thick black line.

The ROC curve above describes the average performance of a set of samples. We also wished to understand the performance of individual samples. As can be seen in Figure 3, individual samples have different false positive rates given the same sensitivity level.

**Figure 4:**
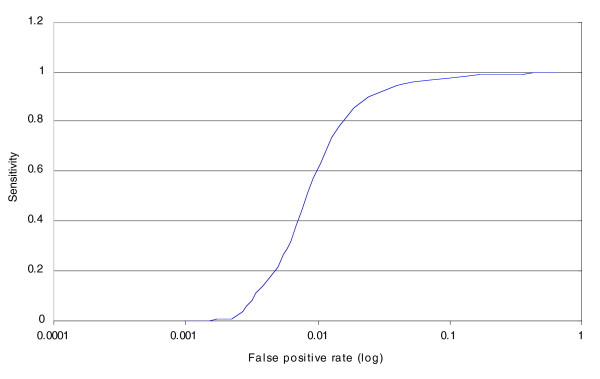
ROC analysis for allele ratio. The x-axis is the rate of false positives (in log_10_), computed as the proportion of autosomal markers that have allele ratio above a threshold. The y-axis depicts sensitivity, defined as the proportion of X chromosome markers in the cell line carrying 3X chromosomes that have copy number values below the same threshold. The curve is generated by calculating these values at many different thresholds.

Similarly, ROC curves can be generated to assess the ability to study ACN. For example, Figure 4 depicts the ROC curve to assess the ability to discriminate the usual 1:1 ratio in heterozygotes from the 2:1 ratio on the X chromosome in a cell line carrying 3X chromosomes. The ROC curve for allele ratio is not as good: at a sensitivity level of 50%, the copy number false positive rate is approximately 1 × 10^-3^, and the allele ratio false positive rate is approximately 8 × 10^-3^. One reason for this discrepancy is that we are using the best markers as defined by copy number root square deviation. The use of the best markers as defined by an allele ratio criterion (allele ratio root square deviation) significantly improves the performance (sensitivity of 50% and false positive rate of approximately 3 × 10^-3^.

#### Systematic false positives

The above analysis assumes that all the autosomal markers are present at two copies per cell. There has been a wealth of evidence demonstrating copy number polymorphisms (CNPs) in the general population [14,15]. Therefore, a fraction of what we considered as false positives may in fact be true positives. In addition, the presence of a secondary SNP (distinct from the one being interrogated) within the probe may emulate the presence of a deletion. Data generated on two CEPH pedigree populations, Yoruban and Utah, are informative in this regard because the polymorphisms on which the MIP panel is based are from European (equivalent to Utah) rather than African populations. The contribution of genetic variants (CNP or SNP) to the apparent false positive rate is suggested by our detection of approximately three-fold more apparent autosomal deletions in the Yoruban population compared to the Utah population (average number of markers per sample with measured copy number below 1.3 is 126 markers for the Utah population and 319 for the Yoruban population). We hypothesized that this imbalance between the number of apparent deletions in the two populations was likely due to secondary polymorphisms close to the SNP being assayed, which prevent proper binding of the MIP to its target. Further evidence to support this hypothesis was noted when we observed that the majority of these apparent deletions were reproducible when a sample is re-assayed.

To understand the nature of these apparent deletions, we randomly picked nine SNPs, which showed copy number measurements below 1.3 in replicate measurements from the Yoruba sample (sample NA18515). We PCR amplified approximately 400 base-pair fragments that included the SNP assayed by MIP and used dideoxy sequencing to show that eight of these nine loci that were successfully sequenced had a secondary SNP within the MIP probe homology sequence. The ninth SNP that showed copy number 1 was assayed by qPCR to measure copy number but was found to show a normal copy number of two (Supplementary Table 1 in Additional data file 1).

#### Trade off between resolution and performance

Copy number changes are expected to occur in discrete segments, allowing neighboring markers to be averaged together. This leads to enhanced performance as measured by the trade off between false positive rate and sensitivity (that is, the ROC curve moving to the upper left) at the expense of lower resolution.

As discussed above, one shortcoming of the ROC analysis is the presence of CNPs in the autosomes. Averaging two adjacent markers that lie within a CNP will erroneously consider these markers as false. Therefore, for the purpose of describing the performance of the technology, we averaged markers that are not adjacent to each other. This method would ameliorate the effect of miscalling two adjacent markers in a CNP as a false positive. This analysis is appropriate as long as there is a lack of correlation between marker performance and the position on the chromosome. If this assumption is true, then the operation reflects the performance of averaging two adjacent markers since the adjacent and the random markers are obtained from the same distribution. Clearly, averaging data from non-adjacent markers is valid only for the assessment of the technology performance and cannot generate any meaningful biological findings.

**Figure 5:**
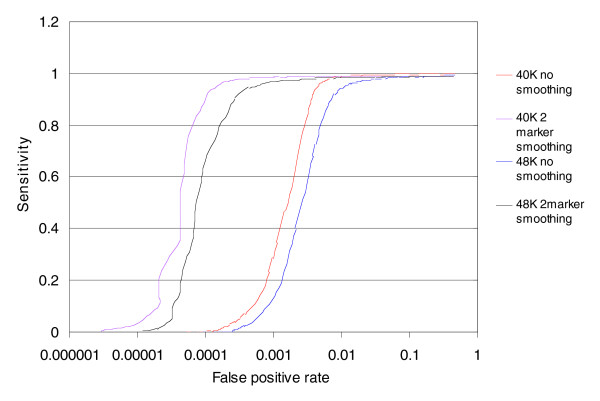
ROC analysis for two-marker smoothing. The same ROC analysis as described in Figure 2 was performed here using the same set of markers (~40 K) as well as using a larger number of markers (~48 K). The ROC analysis was also performed using two-marker smoothing. In this case the smoothing was done for two random markers. If we assume that the performance of individual markers is not correlated with their position (that is, markers close together are likely to have similar performance), then this should be an accurate reflection of the resultant performance with adjacent marker smoothing. We note that at the lower false positive rate for the two-marker smoothed data, the curve is not smooth given low statistics.

Averaging over two markers improves the performance of the MIP data significantly (Figure 5). Clearly, when one is trying to obtain biological information, smoothing non-adjacent markers is totally erroneous. In this case we were interested in the exact opposite: erasing any real biological information (copy number polymorphisms) and, hence, we smoothed across non-adjacent markers. For the discrimination between 1 and 2 copies, a sensitivity level of 80% and a false positive rate of 5 × 10^-5 ^can be achieved.

The ROC curves shown in the above figures describe the performance of the top approximately 75% of the markers in the panel we constructed. It is expected that as more of the lower quality markers are considered, the ROC performance will decrease. We included approximately 48,000 markers (approximately 90% of the total) in the analysis. Figure 5 shows the ROC curve to discriminate one from two copies using one marker or two markers using 75% (40 K) or 90% (48 K) of the data. As can be seen in Table 1, the average performance with 90% of the markers is somewhat worse than that seen with 75% of the markers when judging the specificity at 50% sensitivity.

**Table 1:** Sensitivity at 50% specificity

	One marker	Two markers
40 K (75% of data)	1.7E-03	4.0E-05
48 K (90% of data)	2.7E-03	7.1E-05

### Accuracy of copy number estimation

The ROC curves describe the discrimination between two copies and a specific aberration. However, they do not define the accuracy of the copy number estimation. The accuracy of the copy number determination can be estimated by the deviation from the true copy number. This can be readily measured for one to five copies using the X chromosome series. As can be seen in Table 2, the copy number estimation in the MIP data is very close to the true value. The precision, as defined by the relative standard deviation, over the one to five copy number range is 0.1-0.14.

**Table 2:** Expected versus measured copy number

Expected copy number	Measured copy number	Relative standard deviation
1 (9)	1.055	0.14
2 (15)	1.997	0.12
3 (2)	3.104	0.11
4 (2)	3.981	0.10
5 (2)	4.956	0.10

Values in Parentheses represent the number of replicates measured.

**Figure 6:**
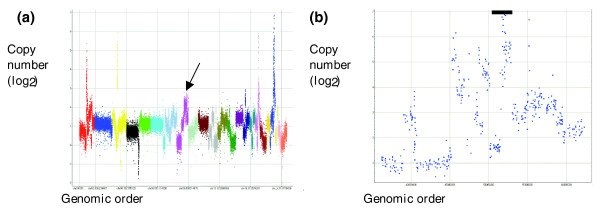
Amplification in MCF7. **(a) **The x-axis shows the markers in a genomic order, with each chromosome uniquely colored. The y-axis depicts the measured copy number for each marker in log_2 _(the log scale is used given the high dynamic range). The arrow depicts the position of the locus that was also analyzed by real time PCR. **(b) **Focused view around the amplification site that was checked with real time PCR. As can be seen, there are several sites of amplifications of different levels. The black bar identifies the region for which average copy number was calculated.

Accuracy of copy number estimation at high copy number amplification can be assessed by comparing the MIP estimation with real time PCR measurement. We have done such a calibration for a selected amplification in cell line MCF7 (Figure 6). The average copy number estimate among 30 MIP markers within the amplification is 43, which is close to the 33 copies measured by real time PCR. Copy number estimation is computed relative to a 'control' region in the genome. In cancer cell lines, the 'control' region used in real time PCR may not have the average ploidy of the cell and, therefore, may bias the estimation of the amplified region. In fact, in this example the control region was from chromosome 2, which is estimated to be present at slightly elevated copy numbers compared to the average of the genome based on the MIP data. Correcting for this bias would make the MIP and real time PCR copy number estimation of the amplification even closer.

**Figure 7:**
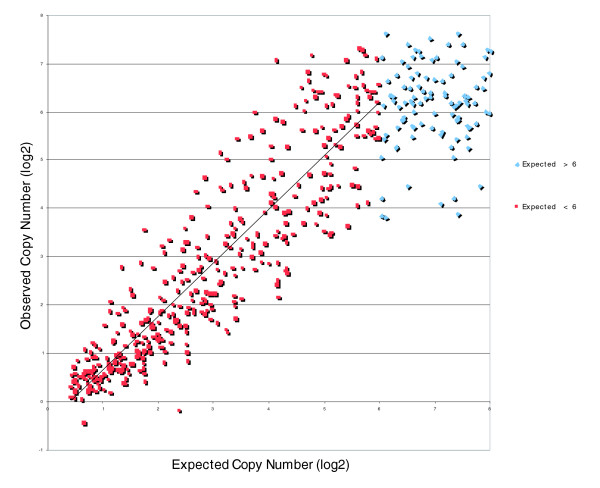
Estimation of copy number of the spikes. The x-axis shows the expected copy number (in log_2_) for the individual spiked in PCR fragments, and the y-axis shows the observed copy number for the same spiked in fragments. The linear fit (r^2 ^= 0.82) is only for spikes with expected copy number <64 (2^6^) because of the clear saturation above that point.

To carefully assess the accuracy of the measurement at high copy number values, we added a known quantity of a set of PCR amplicons to a normal sample before the MIP reaction was performed. The DNA fragments that were spiked in were added at different copy number levels ranging from no extra copies to several hundred additional copies. Supplementary Table 2 in Additional data file 1 shows the PCR amplicons, the MIP probes they correspond to, and the spike in levels. We show the relationship between the expected and the measured copy number of the individual spikes in Figure 7.

The accuracy of measurement of ACN in amplification sites for many methods is limited by allele cross talk. Allele cross talk is the proportion of signal measured for one allele in the presence of a second allele. To assess this phenomenon using MIPs, we studied the spike in data. The spiked in PCR amplicons were purposely generated from an individual that is homozygous and added into DNA from a heterozygous individual, making the copy number for one allele 1 and the other ranges from 1 to 1,000. The allele cross talk in the MIP assay is very low, as the presence of 100 copies or more of one allele does not change the copy number of the other allele significantly (Table 3).

**Table 3:** Allele copy number in spiked samples

Copy_A	Copy_B
199.2	1.3
184.8	1.1
169.4	1.5
141.8	1.3
139.7	0.8
105.4	1.0
84.6	1.0
80.2	0.9
73.8	0.8
73.0	0.9
70.6	1.1
64.5	1.0
60.8	1.0
59.8	1.3
57.4	0.8
54.6	1.1
52.8	1.0
43.3	0.8
39.2	1.1
38.8	0.9
33.4	0.8
27.5	0.8
25.7	0.9
18.7	1.2
14.3	0.7
12.9	0.9
11.3	1.0

### Identification of LOH without matched normal tissue

A major challenge in the study of ACN is the absence of matched normal tissue for many valuable clinical samples. In tumors that have lost one allele, it is not easy to discriminate LOH for individual alleles that are homozygous in the entire individual. We recognized that the high sensitivity and accuracy of the MIP ACN assay, coupled with the high likelihood of normal tumor contamination, could allow us to distinguish LOH from alleles that are homozygous. In theory, this should be best accomplished with tumor showing substantial (approaching 50%) normal contamination.

**Figure 8:**
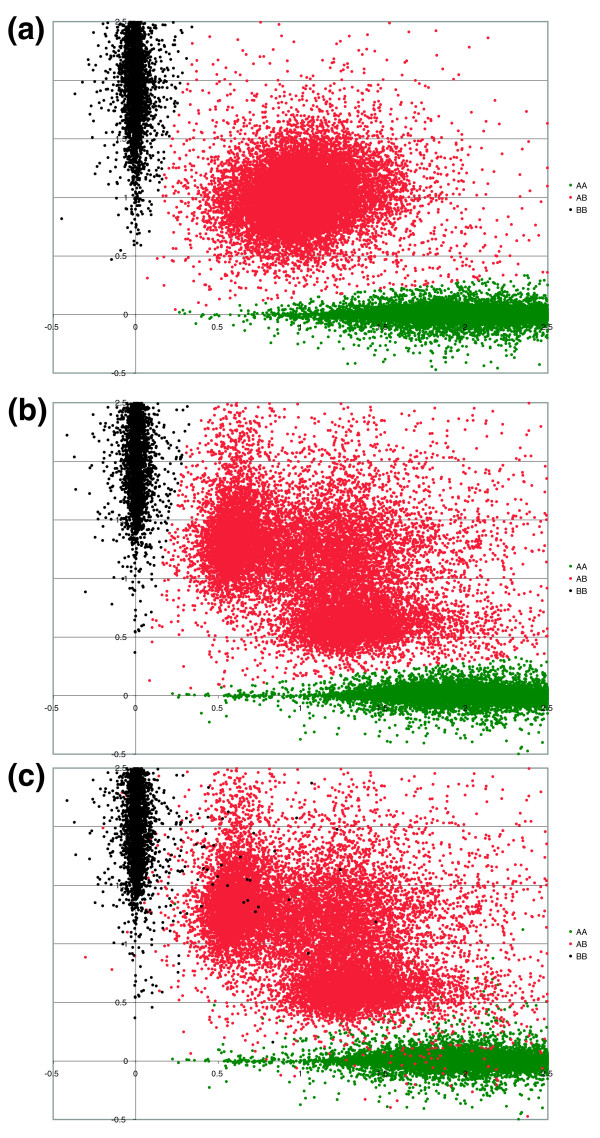
ACN distributions and reproducibility. **(a) **Copy number measurements for tumor sample 47 (fairly normal genome content) with genotypes AA colored red, AB colored blue, and BB colored green. **(b, c) **Allele copy number measurements for tumor 45 (replicate 1). (b) Genotypes derived from replicate 1 are colored AA red, AB blue, and BB green; (c) the genotypes from replicate 2 in the same color scale.

**Figure 9:**
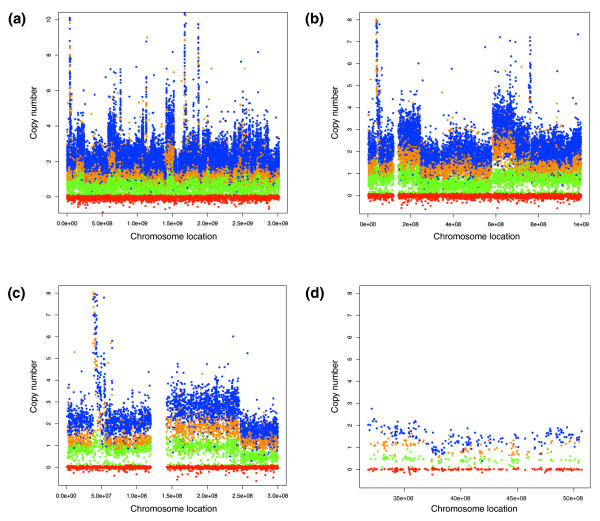
Visualization of individual copy number measurements without matched normal samples. **(a-c) **Copy number measurements for tumor 48 in genome order from chromosome 1 on the left to chromosome 22 and X on the right. Data are segregated by higher and lower copy number estimates and by homozygosity or heterozygosity. Blue and orange data points are the higher allele copy measurement while green and red data points are the lower copy number measurements. Blue and red data points are homozygous alleles while orange and green are heterozygous alleles. (a) The entire genome. (b) Chromosome 1 through the first 100 Mbp of chromosome 5. (c) Chromosome 1 and the first 50 Mbp of chromosome 2, showing key features of ACN data: an amplification is seen near position 5e7; an extra copy of 1q is seen between ~1.5e8 and 2.5e8; a deletion of 1 copy is seen on the p arm of chromosome 2 between ~2.5e8 and 3e8 (observed in (b) as a complete loss of one copy of chromosome 2). **(d) **A small section of chromosome 5 from tumor 44. One chromosome is at copy number 0.5 across this region, which indicates a loss of that chromosome. The black arrow shows a region at total copy number 2, which likely includes reduplication of the lost chromosome in the tumor. The red arrow shows a region where both alleles are at copy number 0.5, suggesting a complete deletion. The green arrow shows copy number 1 for the yellow alleles

To test this theory, we analyzed ACN from five breast tumors using the 60 K MIP panel. Visual examination of the data clearly show a typical plot of estimated copy number for allele A versus allele B, compared to a tumor with relatively normal genome structure (Figure 8a). Three clusters are expected in such a plot, one at ~2, 0 (homozygous A), one at 0, ~2 (homozygous B), and one at ~1, ~1 (heterozygous). In the aberrant tumor samples (Figure 8b,c), three distinct clusters can be observed in the heterozygous cluster. The central cluster represents the 'true' heterozygous copy number measurements. The flanking clusters represent LOH of either the A or B allele. These sub-clusters of the heterozygous cluster clearly resolve into discrete copy number segments along the chromosome, as can be seen in Figure 9. We are also able to observe that deletions are observed not as zero copies for each allele, but as about 0.5 copies of each allele (Figure 9d). To assess reproducibility, we analyzed all samples in duplicate and calculated concordance estimates for the various genotypes (Table 4).

**Table 4:** Genotyping disagreements between replicated samples

Sample	Discordant calls	Total calls	Discordant rate
44	643	50,236	1.3%
45	419	50,260	0.8%
46	271	50,250	0.5%
47	258	50,244	0.5%
48	393	50,242	0.8%

## Discussion

We describe in this manuscript significant improvements we have made to the MIP-based measurements of ACN. By increasing the proportion of genomic targets that are hybridized to the MIP probes, we have improved the performance while requiring a smaller amount of DNA. Additionally, for copy number measurements there are substantial advantages in uniformity and robustness when utilizing one-color readouts, especially at high levels of multiplexing. The use of a control sample that is co-hybridized with the test sample in an analogous fashion as used by BAC arrays leads to inferior results compared with the one color readout (data not shown). Presumably, this is because the different dyes have different characteristics of brightness and saturation. We conclude that the effect of the lack of uniformity among the dyes is probably larger in our system than chip-to-chip variation that the control sample co-hybridization is supposed to ameliorate. The improvements achieved from the new protocol as evaluated by ROC curve analysis resulted in a decline in the false positive rate by an order of magnitude, while reducing the input genomic DNA by more than 25-fold. In addition, the dynamic range has been extended with accurate estimation achieved for up to 60 copies.

We evaluated the performance of MIP for ACN measurements using a set of metrics that are broadly useful for all copy number assays. We demonstrate the ability of MIP to detect a single copy deletion or duplication at an allele and total copy number levels using ROC curve analysis. We believe ROC curve analysis provides a rigorous statistical framework for comparing different technologies or different protocols/algorithms of the same fundamental technology. In addition to genuinely improving the technology performance in the ROC curves by the use of better protocol and algorithms, one may apparently improve them by smoothing (Figure 5), or filtering the worst markers (Figure 5) or the worst samples (Figure 3).

We have shown in the single MIP marker analysis that many of the apparent false positives in the discrimination between 1 and 2 copies are due to the presence of SNPs in the genomic sequence that are complementary to the MIP probes. This effect will be strongest in populations that are the most diverse. It should be possible to ameliorate this effect by using matched normal and tumor pairs. The presence of SNPs may explain why the discrimination between 1 and 2 is not better than that between 2 and 3, as secondary SNPs that interfere with MIP binding emulate a copy number deletion.

We also show the MIP assay precision of measurements of copy number at allele and total copy number levels. Precision at the total copy number level requires low background of the assay and lack of saturation. In addition, allele level precision requires a low level of allele cross talk even when one allele is present in huge excess relative to the other.

These observations led us to suspect that it should be possible to genotype mixed DNA populations, such as occurs in tumor samples contaminated with normal tissue. As normal contamination increases, some estimate of the amount of normal contamination is valuable, which we believe can be quite accurately estimated using the calculated copy numbers for regions of LOH and deletion.

One promise of ACN data over the traditional total copy number data is the potential that it may facilitate the identification of the critical genes in regions of aberrations. Even though large aberrations can be readily identified by total copy number CGH, the identification of the critical gene(s) in these aberrations is often not straightforward. This is in contrast to sequencing data where identification of mutations has been quite laborious, but once achieved the critical gene is usually easily identified. Identification of an allele that is preferentially deleted or amplified in a set of samples implicates the specific allele (or one in linkage disequilibrium with it) as critical in the pathogenesis of the aberrations.

## Materials and methods

### Samples and MIP assay

The normal samples as well as the samples carrying 3 (NA04626), 4 (NA01416), and 5(NA06061) copies of the X chromosome were obtained from Coriell Cell Repository (Camden, NJ, USA). The normal HapMap samples that were used were also obtained from Coriell Cell Repository. The samples that were used were: NA19240, NA19239, NA06991, NA06985, NA19238, NA19222, NA19202, NA19201, NA19200, NA19132, NA19131, NA18956, NA18951, NA18949, NA18947, NA18945, NA18912, NA18854, NA19130, NA19128, NA19127, NA19099, NA19094, NA18991, NA18987, NA18981, NA18605, NA18603, NA18582, NA18573, NA18558, NA18550, NA18547, NA18542, NA18537, NA18515, NA18508, NA12892, NA12813, NA12717, NA12156, NA12155, NA12004, NA11881, NA11840, NA11832, NA11830, NA10831, NA07345, NA07056, NA07029, NA07019, NA07000, and NA06993. The MCF7 cell line was obtained from the American Tissue Cell Culture (ATCC, Manassas, VA, USA).

The MIP assay was performed as described previously, but with important modifications [10]. Specifically, the current protocol is a modification of the targeted genotyping protocol commercialized by Affymetrix (additional information about MIP technology can be found at the Affymetrix website [16]). Briefly, test DNA samples were diluted to 16 ng/ml. All DNA quantification was done using PicoGreen dsDNA Assay Kit (Molecular Probes/Invitrogen, Carlsbad, CA, USA, P7589). We used 96- or 384-well plates whenever possible to reduce variation. For day1 overnight annealing, 4.7 μl of DNA samples (75 ng total), 0.75 μl of Buffer A, 1.1 μl of the 53 K probe pool (200 amol/μl/probe) and 0.045 μl of Enzyme A were mixed well in a 384-well plate on ice. The reaction was incubated at 20°C for 4 minutes, 95°C 5 minutes, then 58°C overnight. On day 2, 13 μl of Buffer A was added to each well with 1.25 μl of Gapfill Enzyme mix. Then, 9 μl of this was put in each of two wells in a 96-well plate. MIP probes were circularized with 4 μl of dinucleotide (dATP with dTTP, dCTP with dGTP) and mixed at 58°C for 10 minutes. The uncircularized probes and genomic DNA were eliminated by addition of 4 μl of Exonuclease Mix and incubation at 37°C for 15 minutes, followed by heat-killing of enzymes. The circularized probes were linearized by the addition of Cleavage Enzyme Mix at 37°C for 15 minutes, then subjected to universal primer amplification for 18 cycles at 95°C for 20 s, 64°C for 40 s and 72°C for 10 s. For the labeling reaction, the product was further amplified with the label primers for 10 cycles, and then subjected to cleavage by Digest Enzyme Mix at 37°C for 2 h. To hybridize, the cleaved MIP products were mixed with hybridization cocktail, denatured and hybridized to 70 K Universal Taq arrays at 39°C for 16 h (two arrays per sample). The overnight hybridized arrays were washed on GeneChip^® ^Fluidics Station FS450 and stained by SAPE at 5 ng/ml (Invitrogen).

Copy number estimation was obtained from the hybridization signals as described previously, but with the following modifications [10]. Given that in this work no multi-color readout was present (but rather single color readout on two arrays), no spectral overlap was present and, therefore, the color-seperation step was omitted. In addition, instead of the linear calibration of the allele signals, Langmuir correction was done [13].

### Generation of spike-in samples

A panel of 80 PCR products representing genomic regions containing MIPs on chromosome 2 were PCR amplified from CEPH1341.14 (NA06985) using an ABI 9700 thermocycler (initial denaturation of 95°C for 5 minutes, 95°C for 30 s, 58°C for 30 s, 72°C for 60 s for 30 cycles; final extension at 72°C for 7 minutes). The products were purified using a MinElute 96 UF PCR Purification plate (Qiagen Valencia, CA, USA) and resuspended in TE. The purified products were quantified on a fluorometer using the Quant-It™ dsDNA Assay kit (Invitrogen). Purified PCR products were then pooled into ten tubes, each containing eight different products (Supplementary Table 2 in Additional data file 1). Each pooled tube of probes was then serially diluted two-fold into a series of spike-in tubes containing 150 ng of genomic DNA from CEPH1341.02 (NA06991) (Supplementary Table 2 in Additional data file 1). The genomic DNA samples were chosen so that the spike-in PCR products from CEPH1341.14 represented a single allele, while the genomic DNA from CEPH 1341.02 was heterozygous, allowing for discrimination of allele specific amplification.

### Sequence analysis of aberrant MIPs

PCR products were amplified using primers designed to span sequences containing MIPs that did not hybridize as expected (Supplementary Table 1 in Additional data file 1). Amplification was carried out in a 50 μl reaction (initial denaturation of 95°C for 5 minutes, 95°C for 30 s, 58°C for 30 s, 72°C for 60 s for 30 cycles; final extension at 72°C for 7 minutes) and products were purified using a MinElute 96 UF PCR Purification plate (Qiagen) and resuspended in TE. The purified products were sequenced using an Applied Biosystems (Foster City, CA, USA) 96 capillary 3730 × l DNA Analyzer and the forward and reverse primers used during amplification.

### Identification of LOH without matched normal tissue

Genotyping metrics from the traditional MIP method were applied to each observation and estimated genotypes (AA, AB, or BB) were determined for each MIP in each of five replicated tumor samples. Data are provided as Additional data file 1. Regions of the genome that show clear evidence for decreases in copy number are easily observed with the decrease in copy number equivalent to 1.5 total copies (1 copy of 1 allele and 0.5 copies of the other, or for homozygous alleles 1.5 total copies). No regions of the genome in any of the five samples analyzed appear to have ~1 copy of the higher allele and ~0 copies of the lower allele.

## Abbreviations

ACN, allele copy number; BAC, bacterial artificial chromosome; CGH, comparative genome hybridization; CNP, copy number polymorphism; LOH, loss of heterozygosity; MIP, molecular inversion probe; ROC, receiver operator characteristic; SNP, single nucleotide polymorphism.

## Authors' contributions

YW performed the MIP assays. LE provided DNA samples. NJW and KC generated DNA samples for analysis. MM, JI, and SL developed analytical methodology. MM, LMH, and PTS performed data analysis. PTS and MF wrote the manuscript. LMH and JWG edited the manuscript.

## Additional data files

The following additional data are available with the online version of this paper. Additional data file 1 lists replicated ACN data for five breast cancers. Data are filtered to use only high quality MIPs (90% or greater call rate, less than 12% copy number variation).

## Supplementary Material

Additional data file 1 Data are filtered to use only high quality MIPs (90% or greater call rate, less than 12% copy number variation). Click here for file
